# A colorimetric amplification-based method for identification of *Moraxella catarrhalis*, a human respiratory tract pathogen

**DOI:** 10.1016/j.bbrep.2026.102488

**Published:** 2026-02-12

**Authors:** Kiana Gholizad Monavari, Hamidreza Mollasalehi

**Affiliations:** Department of Microbiology and Microbial Biotechnology, Faculty of Life Sciences and Biotechnology, Shahid Beheshti University, Tehran, 1983969411, Iran

**Keywords:** *M. catarrhalis*, Respiratory infections, Molecular identification, *ompCD* gene, Colorimetric detection

## Abstract

*Moraxella catarrhalis* is a significant pathogen associated with both upper and lower respiratory tract infections. This study aims to identify *M. catarrhalis* using the *ompCD* gene, which encodes a major outer membrane protein. Spectrophotometry was used to evaluate the accuracy of the colorimetric method by measuring the A450/A570. The colorimetric measurements and PCR method specificity were assessed utilizing twenty bacterial samples. The results of *M. catarrhalis* detection confirmed the presence of a specific band at 273 base pairs and a light red color, with an A450/A570 ratio of 1.5. However, 100% specificity was observed in negative samples with an A450/A570 ratio of 1.15 or less, lacking an amplicon band and showing a deep red color. Furthermore, the selectivity of the techniques was confirmed. The methods revealed a detection limit of 0.05 *n*g/*μ*L. The colorimetric PCR approach is a suitable point-of-care test for rapidly and accurately identifying *M. catarrhalis*.

## Highlights

• First-time detection of *Moraxella catarrhalis* using the *ompCD* gene colorimetric approach.
• A colorimetric PCR technique was optimized to detect *M. catarrhalis* both by the naked eye and quantitatively.
• To examine the diagnosis, endpoint spectrophotometry at 450 and 570 nm was carried out.
• The specificity and selectivity of the method were investigated using several bacterial and mixed samples.
• A sensitivity of 0.05 *n*g/*μ*L and 100% specificity was achieved.

## Graphical abstract

**Figure:**
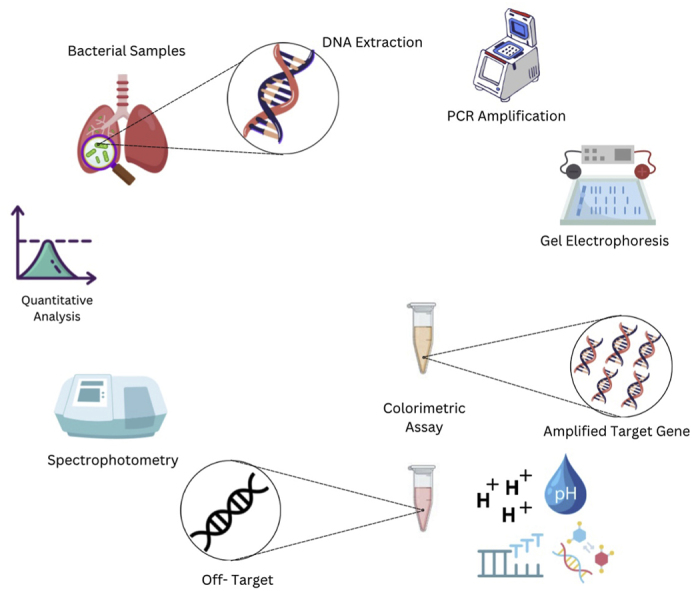

## 1 Introduction

*Moraxella catarrhalis* (*M. catarrhalis*), a Gram-negative, non-capsulated, and aerobic diplococcus, was regarded as a component of the upper respiratory tract flora [1,2]. It has become a major human pathogen causing several respiratory and other diseases ([3]). *M. catarrhalis* has been identified as a nosocomial pathogen and a significant, prevalent respiratory infection in people since the late 1970s [[4], [5], [6]]. Besides the increasing frequency of beta-lactamase-producing strains, *M. catarrhalis* has generated a new interest in the bacterial species [[7], [8], [9]]. Since the 1970s, resistance among *M. catarrhalis* strains has gradually risen. 90 to 100 percent of *M. catarrhalis* strains generate beta-lactamase ([10,11]; Tille and BA, 2017). This mucosal pathogenic bacterium causes acute otitis media (AOM) and sinusitis in children, and chronic obstructive pulmonary disease (COPD) in elders [10,12,13]. *M. catarrhalis* contributes 10% of adult COPD bacterial exacerbations [6]. It is responsible for around 20% of acute bacterial sinusitis cases in children, whereas in adults, it accounts for a smaller share of sinusitis [14,15]. About half of kids have at least one type of AOM before they are 1 year old, and by age 3, that figure reaches 80% [7,16,17]. Potential hearing loss from AOM puts children at risk of speech and language development delays; unfortunately, no vaccination is available for this bacterium [11,18]. Consequently, *M. catarrhalis* causes recurrent illnesses in children, leading to increased antibiotic use [19]. This bacterium can lead to severe infections in hosts with immunological deficits or impairments, including pneumonia, laryngitis, endocarditis, septicemia, and meningitis [7,20,21]. Older individuals with underlying diseases such as diabetes, cardiovascular illness, and chronic renal disease have a higher risk of serious infections [22]. While *M. catarrhalis* rarely causes pneumonia, some cases have shown that elderly individuals, particularly those with preexisting heart and lung conditions, are more susceptible to infection [15,23].

Diagnosis and treatment of infections depend on accurate and rapid identification of this bacterium, as its clinical symptoms overlap with those of other respiratory diseases and are not readily distinguishable [24]. Identifying *M. catarrhalis* using conventional cultural methods can be difficult due to their limitations [19]. The ideal growth condition for *M. catarrhalis* is incubation at 35 °C to 37 °C for 72 h; however, this extended incubation delays early diagnosis and therapy [25,26]. *M. catarrhalis* colonies also resemble *Neisseria* colonies, which could cause misidentification and may lead to an unsuitable treatment [7,13]. Furthermore, biochemical diagnostic techniques require multiple tests targeting the specific biochemical traits of this bacterium, such as enzyme synthesis [26]. Molecular techniques, including polymerase chain reaction (PCR)-based methods, provide rapid results with high sensitivity and specificity [19,24]. Colorimetric assays were also used to assess the outcomes of the molecular methods [27]. These techniques use several indicators, including metal indicators and pH indicators, as well as nanomaterials such as gold nanoparticles [28,29]. Colorimetric detection reduces diagnostic time and enables real-time detection at lower cost, with fewer tools and faster action [30,31].

This study aims to develop a colorimetric PCR method using specific primers derived from the critical gene *ompCD* for the rapid and specific identification of *M. catarrhalis*. We examined PCR products using a colorimetric method with a pH-sensitive color indicator, enabling patient-centered analysis by the naked eye based on changes in the reaction. The specificity of the assay was evaluated, and its sensitivity and selectivity were assessed.

## 2 Materials and methods

### 2.1 Chemicals and bacterial strains

The Brain Heart Infusion (BHI) culture media was supplied by Ibresco Life Science (Iran), whereas the Tryptic Soy Broth (TSB) and Tryptic Soy Agar (TSA) were sourced from Liofilchem S.r.l. (Italy). Forward and reverse primers were procured from Metabion (Germany). The 2x master mix was procured from Parstous (Iran), the enhancer buffer from Kawsar Biotech (Iran), agar from GenFanAvaran (Iran), Tris-Lab from Merck (Germany), Ethylenediaminetetraacetic Acid (EDTA) from Biochem Chemopharma (France), boric acid from DRM CHEM (Iran), 5x loading dye and 100 base pair DNA size marker from SinaClon (Iran), gel stain from Denagene Tajhiz, and agar acquired from Ghatran Shimi Tajhiz (Iran). All chemicals were of molecular biology grade. Bacterial specimens were acquired from the Iranian Research Organization for Science and Technology (IROST) and the Iranian Biological Resource Center (IBRC) (Table 1).

**Table 1:** Names of the bacteria used in this study.

Referral code	Names of Bacteria
ATCC 25240, PTCC 1928	*Moraxella catarrhalis*
ATCC 10973, IBRC-M 10676	*Moraxella osloensis*
IBRC-M 10633	*Listeria ivanovii*
ATCC 6051, PTCC 1720	*Bacillus subtilis*
IBRC-M 10979	*Brochothrix thermosphacta*
ATCC 15442, PTCC 1707	*Pseudomonas aeruginosa*
PTCC 1151	*Yersinia enterocolitica*
PTCC 1078	*Morganella morganii*
ATCC 25416, PTCC 1869	*Burkholderia cepacia*
ATCC 13880, PTCC 1621	*Serratia marcescens*
ATCC 13932, PTCC 1783	*Listeria monocytogenes*
ATCC 25922, PTCC 1399	*Escherichia coli*
ATCC 6538, PTCC 1112	*Staphylococcus aureus*
ATCC 13048, PTCC 1221	*Klebsiella aerogenes*
ATCC 8090, PTCC 1600	*Citrobacter freundii*
ATCC 29212, PTCC 1778	*Enterococcus faecalis*
PTCC 1609	*Salmonella typhi*
PTCC 1290	*Klebsiella pneumoniae*
ATCC 43071, PTCC 1776	*Proteus mirabilis*
ATCC 9207	*Shigella boydii*
PTCC 1855	*Acinetobacter baumannii*

### 2.2 Bacterial culture media

While 19 distinct species were cultured in 50 mL of TSB at 37 °C for 18 to 24 h, the lyophilized bacteria *M. catarrhalis* and the actively cultivated *M. osloensis* were cultured in 50 mL of BHI Broth at 37 °C for 24 to 36 h. Purity and possible contamination were evaluated using BHI Agar and TSA; DNA extraction was performed from BHI broth and TSB culture media.

### 2.3 Nucleic Acid Extraction

The boiling method was utilized to extract genomic DNA. 500 μL of the medium containing bacteria was placed into a sterile 1.5-mL microtube. In a spinner, the microtube was centrifuged for 5 min at 5590×*g*. The supernatant was disposed of. After that, 500 μL of sterile, deionized water was added to the microtube. A gentle wave of the hand or a low-speed vortex helped dissolve the remaining sediments in the deionized water. The microtube was submerged in hot water for 10 min. The microtube was spun for 5 min at 5590×*g* in the spinner. The DNA supernatant was refrigerated. DNA samples were examined after genomic DNA extraction to confirm the absence of contamination and correct performance for the following directions. Nanodrop spectrophotometry used for this aim, as it measures the absorbance of the DNA sample at 260 nm, which indicates the DNA concentration (Table 2). If the concentration of the DNA samples exceeded 60 *n*g/*μ*L, they were normalized to 45 ng/μL with sterile deionized water. All samples were treated under identical conditions (Supplementary Table 1).

**Table 2:** Information obtained from the analysis of DNA samples using the nanodrop spectrophotometer before the normalization.

DNA concentration	Sample Name
49.8 *n*g/*μ*L	*Moraxella catarrhalis*
131.5 *n*g/*μ*L	*Moraxella osloensis*
48.75 *n*g/*μ*L	*Listeria ivanovii*
35.3 *n*g/*μ*L	*Bacillus subtilis*
25.1 *n*g/*μ*L	*Brochothrix thermosphacta*
34.5 *n*g/*μ*L	*Pseudomonas aeruginosa*
56.4 *n*g/*μ*L	*Yersinia enterocolitica*
42.4 *n*g/*μ*L	*Morganella morganii*
39.1 *n*g/*μ*L	*Burkholderia cepacia*
191.4 *n*g/*μ*L	*Serratia marcescens*
20 *n*g/*μ*L	*Listeria monocytogenes*
16.8 *n*g/*μ*L	*Escherichia coli*
24.05 *n*g/*μ*L	*Staphylococcus aureus*
124.95 *n*g/*μ*L	*Klebsiella aerogenes*
171.9 *n*g/*μ*L	*Citrobacter freundii*
65.5 *n*g/*μ*L	*Enterococcus faecalis*
46.9 *n*g/*μ*L	*Salmonella typhi*
37.2 *n*g/*μ*L	*Klebsiella pneumoniae*
175 *n*g/*μ*L	*Proteus mirabilis*
37.1 *n*g/*μ*L	*Shigella boydii*
94.35 *n*g/*μ*L	*Acinetobacter baumannii*
60.5 *n*g/*μ*L	DNA Mixture of Sputum and *Moraxella catarrhalis*
107.6 *n*g/*μ*L	A mixture of *Moraxella catarrhalis* culture and Sputum
10.4 *n*g/*μ*L	Sputum DNA sample
48.3 *n*g/*μ*L	The Positive Mixture of Bacterial DNA
45.4 *n*g/*μ*L	The Negative Mixture of Bacterial DNA
22.9 *n*g/*μ*L	Positive Bacterial Culture Mix
195.4 *n*g/*μ*L	Negative Bacterial Culture Mix

### 2.4 Target gene selection and primer design

The BLAST tool on NCBI was used to investigate the genes in *M. catarrhalis*. The *ompCD* gene encoding the outer membrane protein (OMPCD) was selected under GenBank accession number AY493741. The Gene Runner 6.5.50 program generated two forward and reverse primers to amplify the target sequence (Table 3). Further investigation on the characteristics of the primers was done using NCBI Primer-BLAST. The BLASTN tool was finally applied to guarantee each primer's specificity.

**Table 3:** Designed primers for the specific identification of *M. catarrhalis.*

Sequences (5′⟶3′)	Length (nucleotide)	Tm (°C)	%GC	Amplicon Length (nucleotide)	Reference
TGGGAAGGCTTAGCGTTGGCTGGTTTAGAGG	31	71.18	54.84		This study
GCAACCGCGTGGATCAACAACAGTGTTTACTGG	33	71.13	51.52	273	This study

### 2.5 PCR and gel electrophoresis

The PCR reaction was performed in a 12.5 μL reaction mixture containing 2.5 μL of sterile, deionized water, 2.5 μL of 1.5 M enhancer buffer, 6.25 μL of 2X master mix, 1 μL of a forward and reverse primer mix at 10 μM, and 0.5 μL of the extracted DNA sample. The PCR mixture underwent a thermal cycling protocol that included an initial denaturation at 95 °C for 5 min, followed by 35 cycles comprising denaturation at 95 °C for 30 s, annealing at 69 °C for 1 min, and extension at 72 °C for 30 s, concluding with a final extension at 72 °C for 5 min in a thermocycler. The PCR amplicons were assessed using gel electrophoresis with visual detection under UV light. To make a 1.5% agarose gel, 0.3 g of agarose powder was dissolved in 20 *m*L of 1X TBE buffer by heating. For sample loading, 2.5 μL of a 100 bp DNA size marker was combined with 0.9 μL of loading dye, while 4 μL of the PCR products were mixed with 0.7 μL of loading dye before being placed into the wells.

### 2.6 Colorimetric assay

For colorimetric analysis, 0.022 g of Neutral Red powder is vortexed into 1500 μL of sterile deionized water. From this Neutral Red solution at 50 *m*M, 25 μL is mixed with 475 μL of sterile deionized water to obtain a final concentration of 2.5 *m*M. The process entails incorporating 11% of the total solution volume of the Neutral Red indicator into the reaction mixture (4 μL of the sample and 0.5 μL of the indicator).

### 2.7 Evaluation of specificity, sensitivity, and selectivity of the assay

The PCR products were evaluated by measuring the absorbance of the samples in the visible spectrum (400-700 *n*m) using a Nanodrop spectrophotometer. The specificity of the assay was assessed using genomic DNA from *M. catarrhalis* and 20 other selected bacteria from related or different genera and species, according to G Power software (Supplementary Table 2) [32].

A diluted, extracted *M. catarrhalis* DNA sample was used to evaluate the sensitivity of the method. The DNA sample was diluted to $10^{-8}$. The ultimate volume was 12.5 μL, with a DNA sample volume of 0.5 μL, and an initial DNA concentration of 49.8 *n*g/*μ*L. The dilution series was prepared by mixing one part of the DNA sample (1 μL) with nine parts of sterile deionized water (9 μL). Additionally, we used a mix of bacterial samples and an artificially contaminated clinical sample to assess the selectivity of the method. As mentioned, after extracting genomic DNA from each bacterium and examining it, a positive bacterial DNA mixture (containing *M. catarrhalis* genomic DNA along with the other 20 bacterial DNA samples) and a negative bacterial DNA mixture (without *M. catarrhalis* genomic DNA) were prepared. In addition, mixed bacterial cultures were made. All bacteria were grown in a single culture medium and incubated at 37 °C for 24 to 48 h. The negative microbial culture mix sample lacked *M. catarrhalis*, and the positive control sample included *M. catarrhalis* and other bacteria. As discussed in the “Nucleic Acid Extraction” section, DNA was extracted using the boiling method. A sputum sample was also collected, and after DNA extraction using the same boiling method, the DNA was mixed with *M. catarrhalis* DNA (an artificially contaminated sample). Extracted DNA from the sputum sample was used as a negative control. In addition to combining nucleic acid materials from *M. catarrhalis* and sputum, the sputum itself was mixed with the culture medium of *M. catarrhalis* bacteria. Afterwards, their nucleic acids were extracted and analyzed.

## 3 Results

We aimed to develop a colorimetric PCR method for detecting *M. catarrhalis* using a nucleobiomarker. By examining the bacterial genome, 28 candidate genes coding virulence factors were analyzed [21,[33], [34], [35], [36]], and the *ompCD* gene was selected due to the high number of target strains with a similarity percentage of ≥ 95% and query coverage ≥ 91%. The colorimetric PCR-based method was optimized for the qualitative and quantitative detection of *M. catarrhalis*. In that case, after accounting for confounding factors, the assay was designed to detect the *M. catarrhalis* target sequence accurately. Visual turbidity examination and measurement analysis were used to clarify the electrophoresis results.

### 3.1 Specificity analysis of the method

**Fig. 1:**
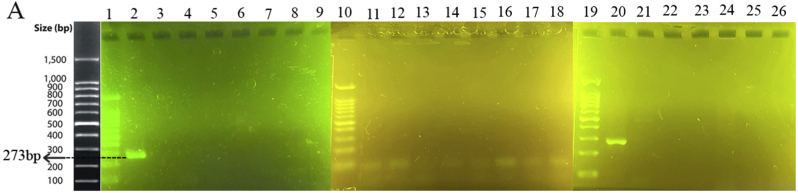

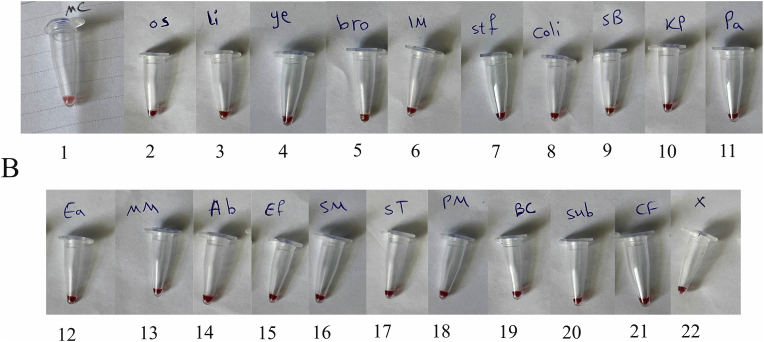

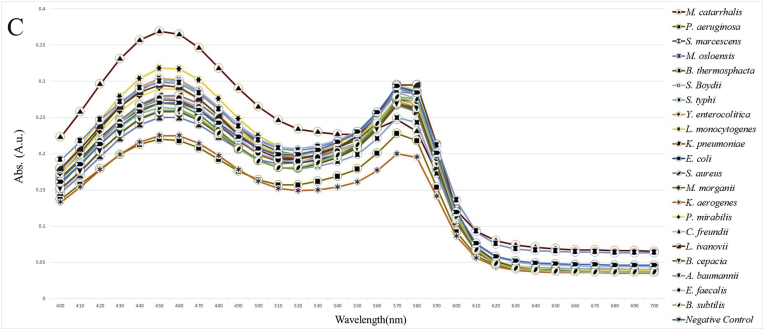
Analysis of detection method specificity. (A) Agarose gel (1.5%) electrophoresis of PCR products. 1: 100 bp DNA Size Marker, 2: *M. catarrhalis*, 3: *M. osloensis*, 4: *L. ivanovii*, 5*: Y. enterocolitica*, 6: *B. thermosphacta*, 7: *L. monocytogenes*, 8: *S. aureus*, 9: *E. coli*, 10: 100 bp DNA Size Marker, 11: *S. Boydii*, 12: *K. pneumoniae*, 13: *P. aeruginosa*, 14: *M. morgani,* 15: *A. baumannii*, 16: *E. faecalis*, 17: *S. marcescens*, 18: *S. typhi*, 19: 100 bp DNA Size Marker, 20: *M. catarrhalis*, 21: *P. mirabilis*, 22: *B. cepacia*, 23: *B. subtilis*, 24: *K. aerogenes*, 25: *C. freundi*, 26: Deionized Water (without DNA template). (B) Colorimetric Evaluation. 1: *M. catarrhalis*, 2: *M. osloensis*, 3: *L. ivanovii*, 4: *Y. enterocolitica*, 5: *B. thermosphacta*, 6: *L. monocytogenes*, 7: *S. aureus*, 8: *E. coli*, 9: *S. Boydii*, 10: *K. pneumoniae*, 11: *P. aeruginosa*, 12: *M. morgani*, 13: *A. baumannii,* 14: *E. faecalis*, 15: *S. marcescens*, 16: *S. typhi*, 17: *P. mirabilis*, 18: *B. cepacia*, 19: *B. subtilis*, 20: *K. aerogenes (*known as *E. aerogenes* before*)*, 21: *C. freundi*, 22: Deionized Water (without DNA template-Negative Control). (C) Quantitative evaluation of the colorimetric method. Visible spectrophotometric analysis between 400 and 700 nm was recorded.

**Fig. 2:**
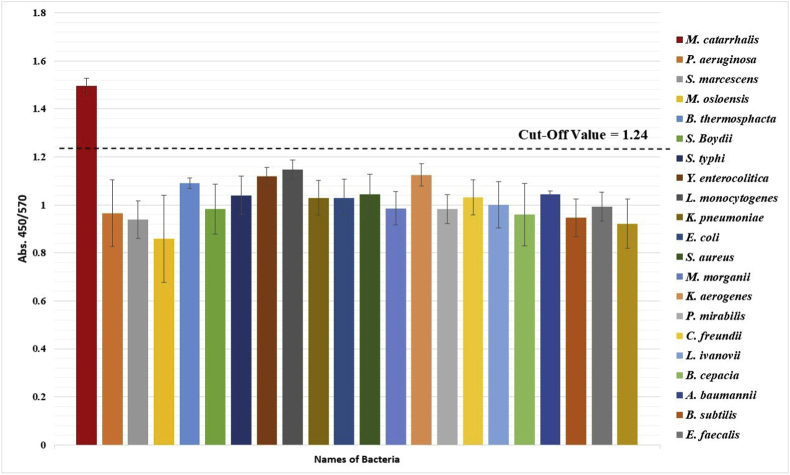
Absorbance values ratio. Comparison of the A450/A570 ratio in *M. catarrhalis* and other bacterial PCR products shows a significant difference (Data are shown as mean ± SD of three independent experiments). The red line represents the cut-off value, calculated as the mean OD of negative samples plus three standard deviations.

The specificity of the method was investigated using both electrophoresis and photometric analysis for various bacteria from related and unrelated species to *M. catarrhalis*. After the amplification stage, the presence of a 273 bp band for the *M. catarrhalis* DNA sample, compared with the absence of the band for the other bacterial samples in the range of 200 to 300 bp, provided evidence for the specificity of the PCR method in this study (Fig. 1A). Additionally, in the photometric evaluation, an endpoint approach was utilized. The colorimetric results indicated that the negative samples appeared light red, while the positive (*M. catarrhalis*) showed a deep red (Fig. 1B). The difference between *M. catarrhalis* and other bacteria was investigated in the visible light spectrum (Fig. 1C). There were two peaks in the absorbance at wavelengths of 450 and 570 nm. All 20 bacteria showed lower absorbance at 450 nm and mostly higher absorbance at 570 nm than *M. catarrhalis*. Figures in the supplementary materials compare absorbance values at 450 and 570 nm wavelengths. Therefore, the A450/A570 ratio was selected as the appropriate indicator of wavelength. The ratio A450/A570 showed a significant difference for *M. catarrhalis* (1.5) compared to the other bacterial samples (≤1.15). Each sample was tested in triplicate, and the mean OD value of replicates was used for further analysis. The cut-off (1.24) was determined as the average of the negative samples plus three standard deviations (Mean + 3SD). Samples with OD values above this threshold were considered positive (Fig. 2). The absorbance values for *M. catarrhalis* at wavelengths of 450 and 570 *n*m are 0.369 and 0.247, respectively. The difference in the A450/A570 ratio between the maximum (*M. catarrhalis*) and the minimum among the bacterial PCR products was recorded as 0.64.

### 3.2 Sensitivity analysis of the method

**Fig. 3:**
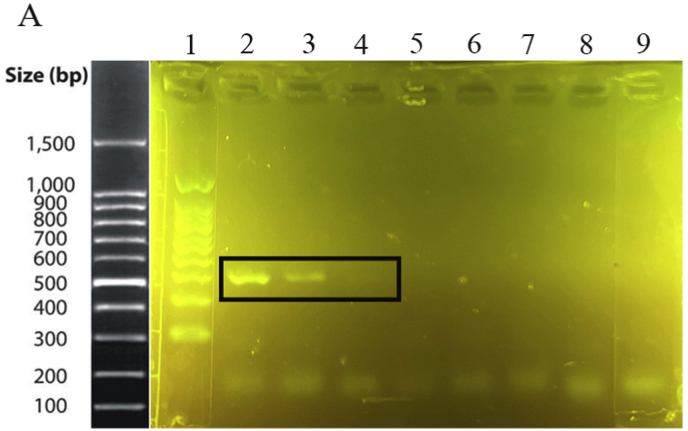

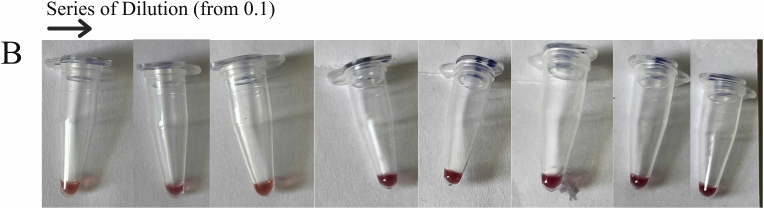

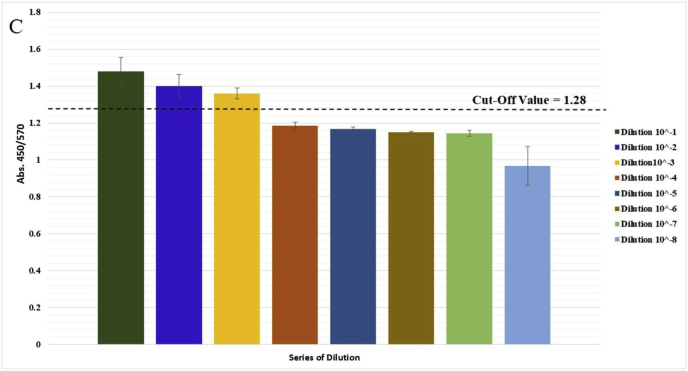
Sensitivity analysis of the PCR method and developed colorimetric assay. (A) Agarose gel (1.5%) electrophoresis of PCR products for sensitivity evaluation, 1: 100 bp DNA size marker, 2: $10^{-1}$, 3: $10^{-2}$, 4: $10^{-3}$, 5: $10^{-4}$, 6: $10^{-5}$, 7: $10^{-6}$, 8: $10^{-7}$, 9: $10^{-8}$. (B) Colored samples of the dilution series are shown, respectively. (C) Quantitative analysis of sensitivity through evaluation of the A450/A570 ratio from spectrometry of colorimetric measurement samples (Data are shown as mean ± SD of three independent experiments). The red line represents the cut-off value, determined as the mean OD of negative samples plus three standard deviations.

The sensitivity of the PCR technique was examined utilizing *M. catarrhalis* DNA as a template. At a dilution of $10^{-3}$, or roughly 0.05 *n*g/*μ*L, the PCR sensitivity data showed a band of 273 bp of the target sequence. On the other hand, the $10^{-4}$ dilution showed no observable band (Fig. 3A). The quantitative evaluation of colorimetric detection revealed that the ratio A450/A570 ranged from 1.48 to 0.97 for dilutions of $10^{-1}$ to $10^{-8}$, respectively. The $10^{-4}$ dilution ratio (1.18) was considered negative. The cut-off value was calculated as the mean absorbance of negative samples ($10^{-4}\;\text{to}\;10^{-8}$) plus three standard deviations (Mean + 3SD), resulting in a cut-off of 1.28. Samples with OD values above this threshold were considered positive (Fig. 3B and C).

### 3.3 Selectivity assessment

**Fig. 4:**
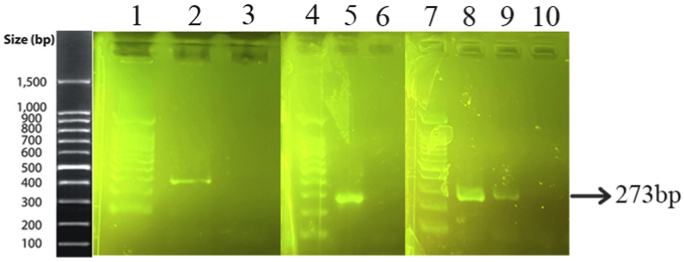
Selectivity analysis of PCR products. Agarose gel (1.5%) electrophoresis of PCR products of mixed and contaminated artificial samples. 1: 100bp DNA size marker, 2: Positive mixture of bacterial DNAs, 3: Negative mixture of bacterial DNAs, 4: 100bp DNA size marker, 5: Positive bacterial culture mix, 6: negative bacterial culture mix, 7: 100bp DNA size marker, 8: DNA mixture of sputum and *M. catarrhalis*, 9: Mixture of *M. catarrhalis* culture and sputum, 10: sputum.

**Fig. 5:**
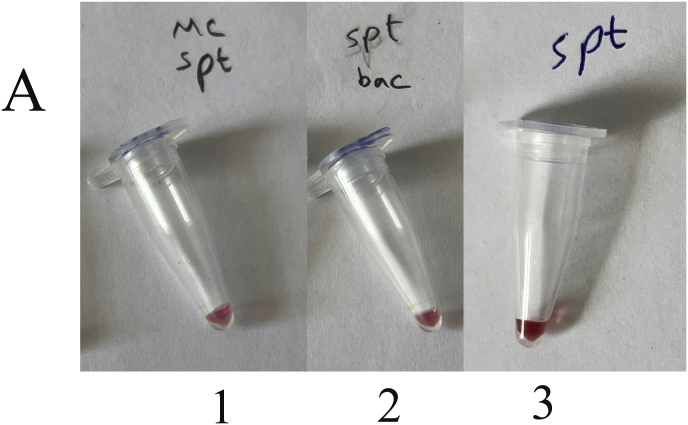

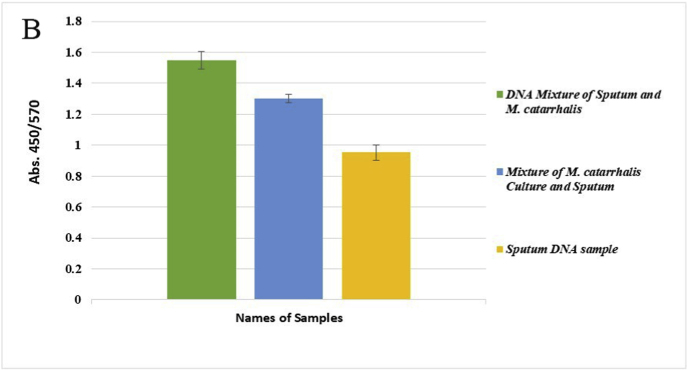

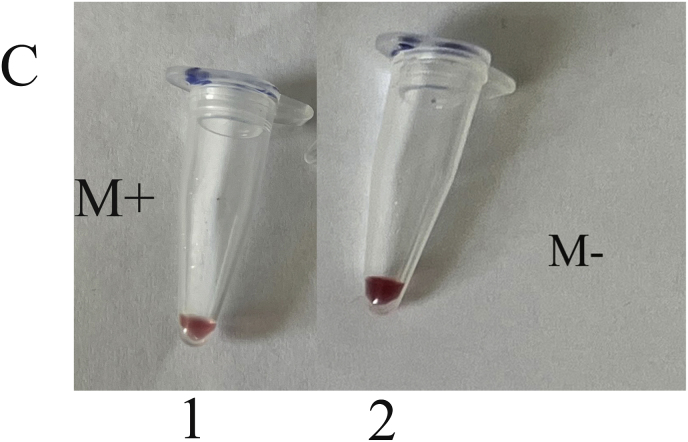

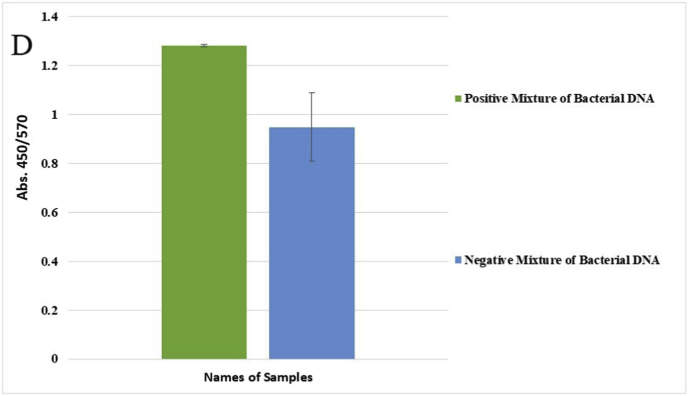

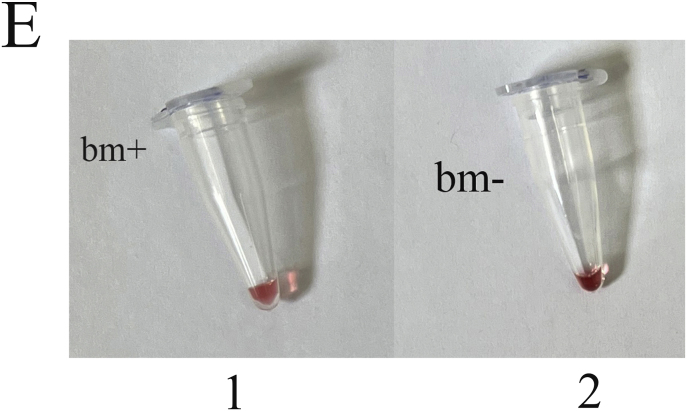

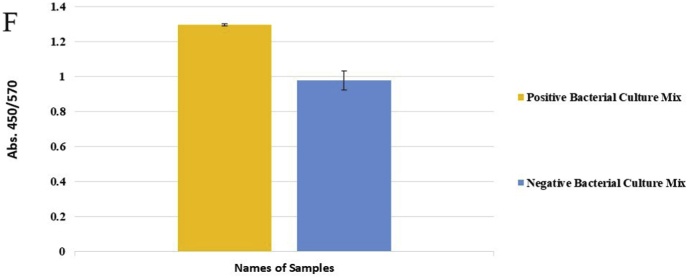
Selective evaluation of the colorimetric assay using visual results. (A) 1: DNA mixture of sputum sample and *M. catarrhalis*, 2: mixture of *M. catarrhalis* culture and sputum sample, 3: sputum sample. (B) The A450/A570 ratio from spectrometry of colorimetric measurements was examined semi-qualitatively, and sputum samples mixed with a positive sample showed similar results to *M. catarrhalis*. (C) 1: Positive mixture of bacterial DNAs, 2: negative mixture of bacterial DNAs. (D) The positive mixed DNA sample display the expected ratio compared to the negative mixed DNA sample. (E) 1: Positive bacterial culture mix, 2: negative bacterial culture mix. (F) The mixed culture medium containing *M. catarrhalis* differs significantly from its negative sample (Data are shown as mean ± SD of three independent experiments).

Genomic DNA from a mixture of bacteria and artificially contaminated sputum samples were used to investigate the selectivity of the method. Amplification of the extracted DNA from the mixed bacterial culture, including a positive sample (*M. catarrhalis*) and the positive microbial culture mixture, produced a 273 bp band. No amplification was observed in the electrophoresis gel for mixed extracted DNAs from negative samples, lacking a positive DNA sample, and the negative microbial culture mix. In addition, the negative DNA sputum sample did not reveal the target band, while the purposely contaminated samples displayed the intended band (Fig. 4). While there was no color change for the negative mixed samples and the negative sputum sample, a color shift happened in the positive mixed samples and the purposely contaminated samples. For the negative mixed extracted DNAs, negative culture mix, and negative sputum sample, the A450/A570 ratios were 0.95, 0.99, and 0.95, respectively. In comparison, the value for the positive mixed extracted DNAs, positive culture mix, mixed DNAs of *M. catarrhalis* and sputum, and mixed sample of sputum with *M. catarrhalis* culture were 1.30, 1.30, 1.55, and 1.30, respectively (Fig. 5A–F).

## 4 Discussion

Due to *M. catarrhalis'* pathogenicity and its role in severe diseases in children, adults, and immunocompromised individuals rapid identification of this bacterium is crucial [7]. Morphological identification and culture-based techniques are insensitive and time-consuming for detecting *M. catarrhalis* [37,38]. However, colorimetric PCR provides faster results without extensive infrastructure, making it especially useful in low-resource settings. Therefore, minimizing unnecessary or excessive antibiotic use and hastening recovery depend on developing rapid and reliable diagnostic techniques. In a study by Post et al. bacterial pathogenic components in middle ear infections were examined by PCR, which found that 46.4% of samples tested positive, compared with 5% with conventional culture methods. They also identified *Haemophilus influenzae* and *Streptococcus pneumoniae* in conjunction with *M. catarrhalis* [39]. In our study, the developed colorimetric PCR method is based on the detection of the *ompCD* gene, which encodes a highly conserved protein crucial for the adhesion process of the bacterium [40,41]. This study provides high-accuracy and high-sensitivity data within a short timeframe (1.5–2 h), demonstrating lower potential errors than conventional biochemical assays. In 2004, Chul-Won Park and colleagues focused on identifying *M. catarrhalis* in pediatric chronic otitis media associated with *H. influenzae* and *S. pneumoniae*. They confirmed the significantly greater sensitivity of PCR for diagnosis, revealing that only 14% of samples tested positive via conventional culture, compared to 36.7% by PCR [42]. Molecular methods, including our study, can also perform identification without a living organism, as only genomic material is required, which is obtained using a simple, low-cost, and rapid extraction method. Farajzadeh et al. (2016) discovered *S. pneumoniae* and *M. catarrhalis* in individuals with chronic paranasal sinusitis using PCR. Among 23 isolates derived from patients, 3.6% were associated with *M. catarrhalis*, which had not been detected by conventional methods [43]. In that regard, in our study, the specificity of the methods was confirmed using a newly designed primer pair, which revealed no cross-reactivity between the PCR and the established colorimetric assay across various bacterial species. In a 2017 study, ear swabs from 50 children with a clinical diagnosis of otitis media were collected and analyzed using conventional methods and PCR. Singleplex PCR was performed using primer pairs targeting the 16 S rRNA gene of *M. catarrhalis*. Conventional methods, including culture, Gram stain, and biochemical tests, showed positive results in 12 (24%) of 50 children (100%) with a clinical diagnosis of otitis media. In comparison, singleplex PCR showed positive results in 10 (83.3%) of 12 samples (100%), indicating that these methods [14] are effective. In addition to standard techniques such as gel electrophoresis, our study developed a visual detection approach. Colorimetric PCR simplifies workflow, making it viable for urgent care. On the other hand, its counterpart, qPCR, often demands expensive thermocyclers and trained personnel. In addition, fluorescence detection may be required for qPCR, whereas colorimetric PCR can be performed with minimal instrumentation. Numerous approaches exist for detecting colorimetric nucleic acid amplification, including probe-based methods and non-specific identification with fluorescent dyes [[44], [45], [46]].

One important event involving the hydrolysis of deoxyribonucleotide triphosphates (dNTPs) is the color change caused by side reactions during PCR, which can lower pH and make the environment more acidic. During oligonucleotide synthesis, the DNA polymerase enzyme produces pyrophosphate (PPi) and adds deoxyribonucleotide triphosphates into the developing DNA strand. Further breaking down pyrophosphate into hydrogen ions and inorganic phosphate (Pi) adds to the acidity of the reaction mixture [47]. As primers bind to the target region and the nucleic acid amplification proceeds, the reaction environment becomes acidic. Neutral red exhibits red coloration under acidic conditions and shifts toward yellow under alkaline conditions. In our experiments, the positive reactions, where amplification occurs, produced a lighter red tone, whereas the negative reactions showed a deeper red color. At first sight, this may appear counterintuitive; however, this pattern is consistent with the complex interactions in molecular samples. The master mix used in this study has a baseline purple color, which may affect the final reaction color. When amplification occurs, proton release lowers the pH and partially shifts the dye toward its acidic form. Because this shift occurs on a purple background, the color appears as a lighter, diluted red-purple rather than an intense, saturated red. In contrast, negative samples do not undergo this proton-induced shift; therefore, the background purple of the master mix combines with neutral red to produce a stronger, more saturated red-purple. Accordingly, this experiment chose a semi-quantitative marker, the A450/A570 ratio. In another study, the aim wavelength was 560 nm, and spectroscopy was performed in a LAMP method to detect SARS-CoV-2 [48] and in a study performed by Sivakumar et al. curcumin was utilized as a natural pH indicator for loop-mediated isothermal amplification of *Staphylococcus aureus* (*S. aureus*) and *Streptococcus pneumoniae* (*S. pneumoniae*) resulting in red-yellow color differentiation [49]. This approach distinguishes between positive and negative samples; this difference is constant and precise even 24 h later. Moreover, this technique offers faster results than usual techniques, such as slow and costly gel electrophoresis.

Mukena Nawa et al. investigated the pathogenicity and microbiological features of *M. catarrhalis* isolates obtained from children with acute pneumonia in 2022. Beta-lactamase strains were identified using morphological and molecular methods. Microbiological methods successfully identified 26.6% of isolates, compared with 100% identification using PCR and DNA sequencing [50]. Thus, the PCR approach is the definitive standard for validation. In our study, in addition to specificity testing across different bacterial species, sensitivity and selectivity were evaluated to demonstrate the method's effectiveness in detecting *M. catarrhalis*. As stronger binding was favored, a slightly higher annealing temperature could improve the specificity. As a result, we designed primers with longer lengths and higher melting temperatures. Moreover, an enhancer buffer was used to reduce non-specific binding and increase enzyme activity [51]. Furthermore, visual inspection in our study achieved high sensitivity, facilitating over-the-counter (OTC) implementation of this technique, and it can detect low amounts of DNA. The capability of the detection method to identify a target among several pathogens or in combination with relevant human samples, similar to clinical samples, is important. A combination of bacteria was used in two ways to assess primer binding to the target gene region, and it was successful in both cases. The mixed bacterial DNA samples were examined to confirm the presence of all bacterial DNAs, and the culture mix samples were analyzed in the culture medium to simulate a growth environment. Similarly, in artificially contaminated samples, a combination of human and bacterial DNAs was used to ensure the presence of DNA from human sputum cells and the bacterial DNA, and a combination of sputum and bacteria-containing medium was used to provide the necessary culture medium for the growth of the target bacteria along with the natural flora. Based on the absorbance ratios, the developed colorimetric PCR method demonstrated acceptable reproducibility across test samples, with at least three repetitions per sample. Along with its advantages, the developed method may also have shortcomings; for instance, the boiling extraction method may not be optimal for all sample types. While alternative extraction methods can provide higher purity, the boiling method was selected for its affordability, accessibility, and simplicity. Additionally, the enhancer buffer may not eliminate non-specific amplification if the reaction is not correctly set up. Additional limitations include potential human error during reagent addition (e.g., Neutral Red), pipetting, and the limited number of patient samples.

## 5 Conclusion

The reliability of PCR as a standard technique was the main driver in selecting the amplification-based technique used in this study. Together with the newly developed colorimetric detection, the speed, accuracy, high sensitivity, specificity, and availability of materials compared to other rapid tests brought us closer to the target of rapid detection of *M. catarrhalis* by the integration and optimization of ompCD-targeted PCR with a straightforward colorimetric readout, which together provide a practical proof-of-concept for diagnostic use. Consequently, this approach may serve as a suitable initial method for investigating and validating the detection outcomes. Considering all these features, this approach could be a means of rapid diagnosis, reducing uncertainties about early prescriptions by providing accessible and reliable laboratory results. Apart from empirical antibiotic treatment, which can also lead to resistance, timely identification helps reduce mortality risk and enables more efficient therapy.

## Declaration

During the preparation of this work, the author(s) used Canva and QuillBot to design a graphical abstract and paraphrasing suggestions, respectively. After using this tool/service, the author(s) reviewed and edited the content as needed and take(s) full responsibility for the content of the published article.

Canva: graphic design platform, https://www.canva.com/templates.

QuillBot: Paraphrasing Tool, https://quillbot.com.

## Availability of data and materials

Data is provided within the manuscript or supplementary information files.

## Funding

There are no funding sources regarding this published article.

## CRediT authorship contribution statement

**Kiana Gholizad Monavari:** Formal analysis, Investigation, Writing – original draft. **Hamidreza Mollasalehi:** Conceptualization, Methodology, Supervision, Validation, Writing – review & editing.

## Declaration of competing interest

The authors declare that they have no known competing financial interests or personal relationships that could have appeared to influence the work reported in this paper.

## Data Availability

Data is provided within the manuscript or supplementary information files.
