# Supplementary material for: A colorimetric amplification-based method for identification of Moraxella catarrhalis, a human respiratory tract pathogen
Source: Biochem Biophys Rep. 2026 Feb 12;45:102488. doi: 10.1016/j.bbrep.2026.102488 (PMC12925048; doi:10.1016/j.bbrep.2026.102488)
Supplement: Multimedia component 1 [file mmc1.docx]

**A colorimetric amplification-based method for identification of *Moraxella catarrhalis*, a human respiratory tract pathogen**

Kiana Gholizad Monavari^1^, Hamidreza Mollasalehi^1^*

^1^Department of Microbiology and Microbial Biotechnology, Faculty of Life Sciences and Biotechnology, Shahid Beheshti University, Tehran, Iran. Postal Code: 1983969411

*Corresponding Author: *Dr. Hamidreza Mollasalehi*

Department of Microbiology and Microbial Biotechnology, Faculty of Life Sciences and Biotechnology,

Shahid Beheshti University, Velenjak, Tehran, Iran.

Tel: +98-21-29905942

Fax: +98-21-22431664

E-mail: [H_mollasalehi@sbu.ac.ir](mailto:H_mollasalehi@sbu.ac.ir)

**Supplementary Materials**

**Supplementary Table 1.** Dilution Results of Some Samples

| Sample Name | Initial Concentration | Dilution Factor | Target Concentration |
| --- | --- | --- | --- |
| *Proteus mirabilis* | *n*g/*μ*L 175 | 0.25 | *n*g/*μ*L 45 |
| *Enterococcus faecalis* | *n*g/*μ*L 65.5 | 0.68 | *n*g/*μ*L 45 |
| *Acinetobacter baumannii* | *n*g/*μ*L 94.35 | 0.47 | *n*g/*μ*L 45 |
| *Klebsiella aerogenes* | *n*g/*μ*L 124.95 | 0.36 | *n*g/*μ*L 45 |
| *Citrobacter freundii* | *n*g/*μ*L 171.9 | 0.26 | *n*g/*μ*L 45 |
| *Serratia marcescens* | *n*g/*μ*L 191.4 | 0.23 | *n*g/*μ*L 45 |
| *Moraxella osloensis* | *n*g/*μ*L 131.5 | 0.34 | n *n*g/*μ*L 45 |
| DNA Mixture of Sputum and *Moraxella Catarrhalis* | *n*g/*μ*L 60.5 | 0.74 | *n*g/*μ*L 45 |
| A mixture of *Moraxella catarrhalis* culture and Sputum | 107.6 *n*g/*μ*L | 0.41 | *n*g/*μ*L 45 |
| Negative Bacterial Culture Mix | *n*g/*μ*L 195.4 | 0.23 | *n*g/*μ*L 45 |

| Exact - Proportion: Difference from constant (binomial test, one-sample case) | | |
| --- | --- | --- |
| Options | α balancing: α/2 on each side | |
| Analysis | A priori: Compute required sample size | |
| Input | Tail(s) | Two |
|  | Effect size g | 0.3 |
|  | α err prob | 0.05 |
|  | Power (1-β err prob) | 0.8 |
|  | Constant proportion | 0.5 |
| Output | Lower critical N | 5.0000000 |
|  | Upper critical N | 15.0000000 |
|  | Total sample size | 20 |
|  | Actual power | 0.8042080 |
|  | Actual α | 0.0413895 |

**Supplementary Table 2.** G Power Data for sample size

**
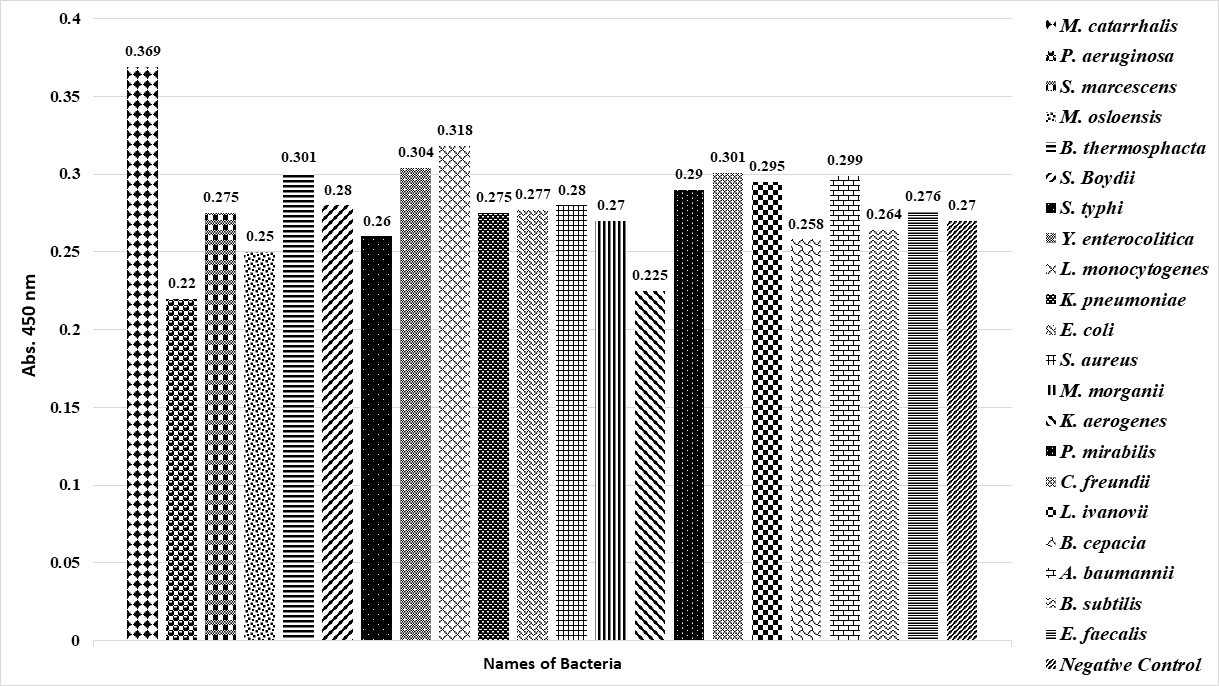
**

**Supplementary Figure 1.** Absorbance values at a wavelength of 450 *n*m. The absorbance value of the positive sample (*Moraxella catarrhalis*) at this wavelength is higher than that of the other samples.

**
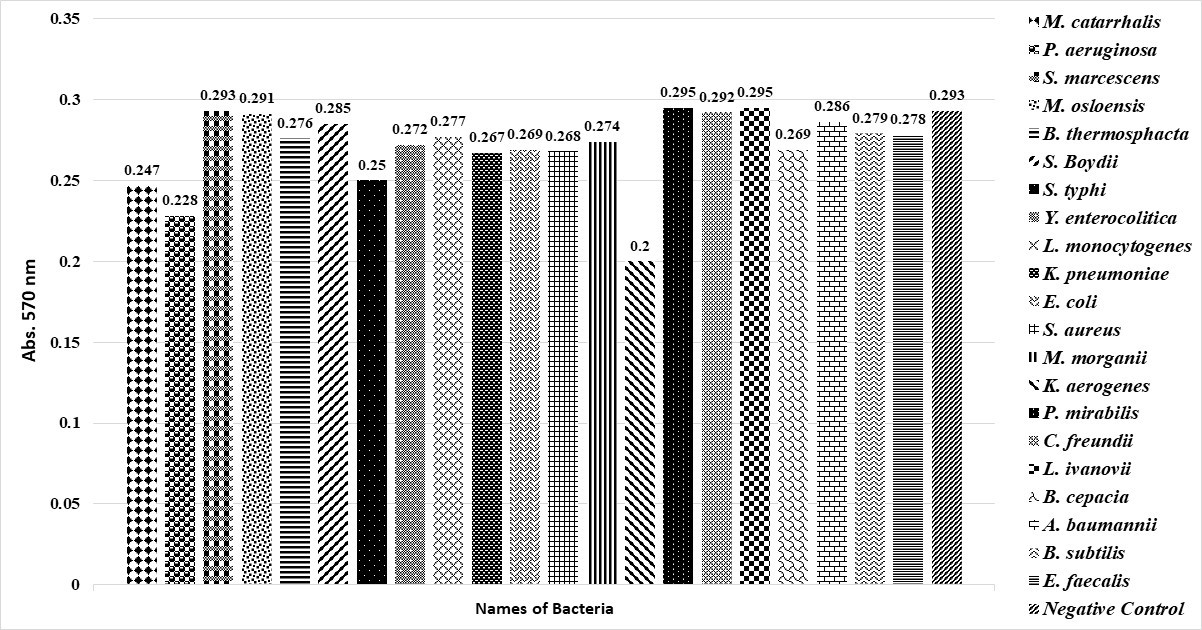
**

**Supplementary Figure 2.** Absorbance values at a wavelength of 750 *n*m. The absorbance of the positive sample (*Moraxella catarrhalis*) at this wavelength is one of the lowest values. Some other samples have low absorption values ​​and an acceptable ratio at the two wavelengths.


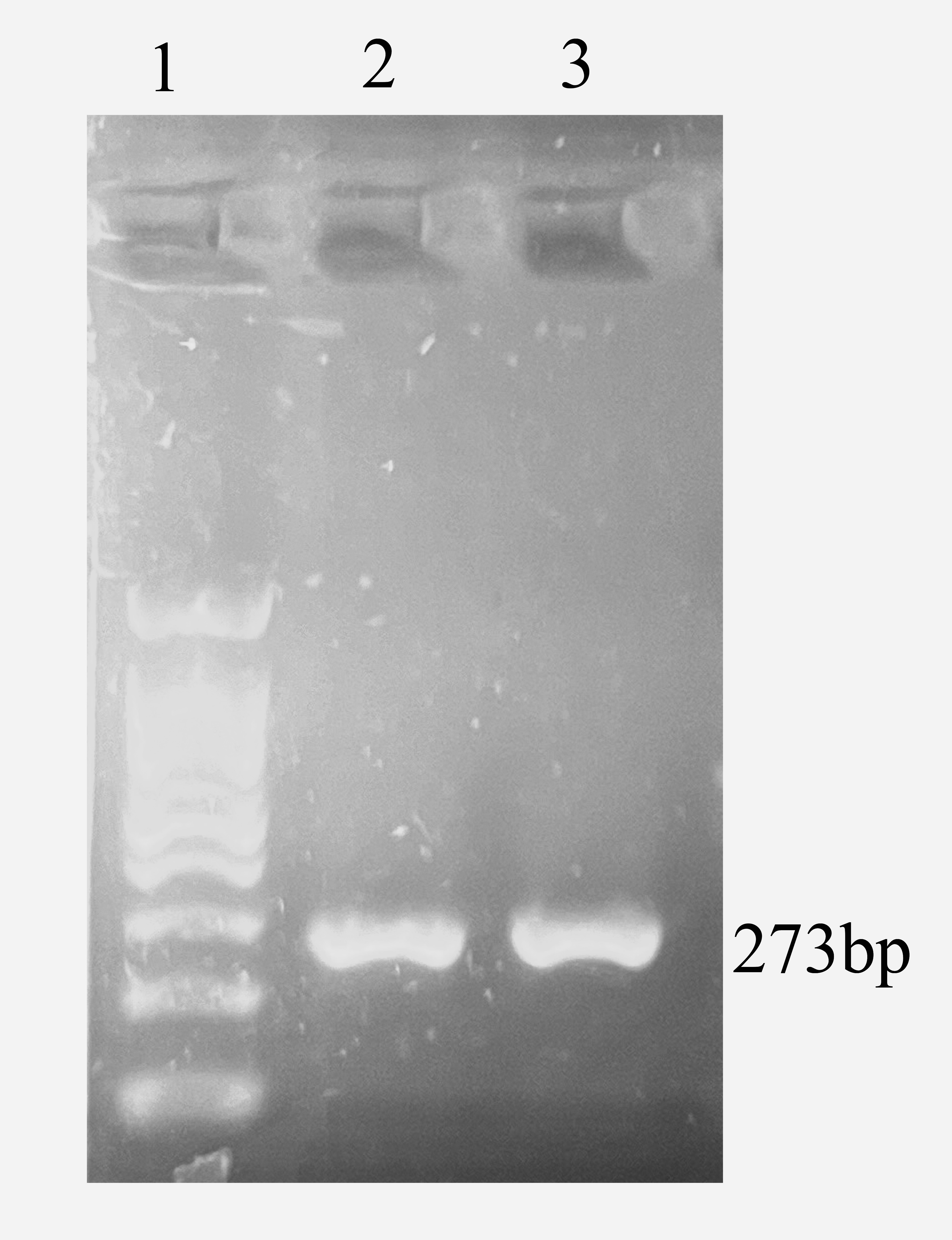


**Supplementary Figure 3.** Agarose gel (1.5%) electrophoresis of PCR products: 1: 100 bp DNA Size Marker, 2 and 3: *M.catarrhalis* (Two different thermal cyclers (DenaGene Tajhiz and Prime).
